# Chronic Bronchitis

**Published:** 1901-10-19

**Authors:** 


### CHRONIC BRONCHITIS.

In the treatment of the chronic bronchitis of the aged one not unnaturally tends to pay attention on the one hand to the particular symptoms of which the patients complain, namely cough, dyspnœa, and difficult expectoration, and on the other to the causes by which, according to the patient, the attacks are principally brought on, namely, cold, fog, and damp weather. Thus it is that the treatment of this form of bronchitis is often directed chiefly in the direction of cough mixtures, stimulating or sedative, as the case may seem to demand, respiratory stimulants and tonics, and various recommendations as to residence, all based on the idea of avoiding the atmospheric condition, the damp and fog, by which the malady seems to be aggravated. It is to be noted, however, that in practically dealing with such cases day by day it often becomes evident there is something more at work than mere weather in producing the attacks, and that a treatment directed to other than the respiratory organs is the best, a liver pill or a dose of potash, or even of the much maligned colchicum, being far more effectual than all the expectorants in the pharmacopœia. The suspicion may indeed be entertained that it is not the coldness of the air in winter that causes winter cough, but rather the change of habits, and the stay-at-home life which the disagreeable weather of the winter leads so many old people to adopt. Dr. Harry Campbell [1] has lately pointed out the important part played by toxis in the production of bronchitis in the aged, and the consequent necessity for curtailing the diet in the treatment of the disease. He says that in the causation of inflammation some chemical irritant is an all but necessary factor, and that such a chemical irritant may be ingested, as in the neuritis of alcohol; or may be produced by microbes, as in pneumonia; or may result from tissue metabolism, as in gout; or finally from defective digestion, especially in the intestines. In the bronchitis of the elderly then we should always keep in view the possibility of intestinal toxicity as a cause of the inflammation. In the treatment of this condition the air breathed should be as pure as possible, which is a very different thing from saying that it should be as warm as possible, and nasal breathing should be insisted on. The diet should, according to Dr. Campbell, be a bare sufficiency, and alcohol and malt should be indulged in sparingly or not at all. Every ounce of superfluous fat should be got rid of, the general health should be maintained at the highest possible level, and breathing exercises should be resorted to with the object of preserving the mobility of the chest walls, while every precaution should be taken against breathlessness by which the elasticity of the lung is apt to be permanently damaged. The arm chair, the warm room, the bronchitis kettle, and the squill and senega mixture are all admirable in their proper place, which is in the treatment of "attacks," exacerbations of the ordinary condition, but they do not tend to cure the chronic bronchitis of advancing life. This is to be prevented by pure air, simple food, exercise, and all those measures which tend to remove those toxic elements, the circulation of which in the blood is the cause of the disease.

[1] Brit. Med. Jour., Oct. 12.

